# Limited evidence for third-party affiliation during development in wild chimpanzees (*Pan troglodytes schweinfurthii*)

**DOI:** 10.1098/rsos.170500

**Published:** 2017-09-13

**Authors:** Jordan A. Miller, Margaret A. Stanton, Elizabeth V. Lonsdorf, Kaitlin R. Wellens, A. Catherine Markham, Carson M. Murray

**Affiliations:** 1 Center for the Advanced Study of Human Paleobiology, The George Washington University, 800 22nd Street, NW, Suite 6000, Washington, DC 20052, USA; 2 Department of Psychology and Biological Foundations of Behavior Program, Franklin and Marshall College, Lancaster, PA, USA; 3 Department of Anthropology, Stony Brook University, Stony Brook, NY, USA

**Keywords:** conflict management, cooperation, third-party affiliation, development, chimpanzee, Gombe National Park

## Abstract

Examining the ontogeny of conflict-mitigating behaviours in our closest living relatives is an important component of understanding the evolutionary origins of cooperation in our species. In this study, we used 26 years of data to investigate the emergence of third-party affiliation (TPA), defined as affiliative contact given to recipients of aggression by uninvolved bystanders (regardless of initiation), in wild immature eastern chimpanzees (*Pan troglodytes schweinfurthii*) of Gombe National Park, Tanzania. We also characterized TPA by mothers in the same dataset as an adult benchmark for interpreting immature TPA patterns. In summary, we found that immatures did not express TPA as measured by grooming between the ages of 1.5 and 12.0 years, and that there was limited evidence that immatures expressed TPA via play. We also found that mothers did express TPA to offspring, although mothers did not show TPA towards non-offspring. Cases of TPA by mothers to other adults were too few to analyse separately. These results contrast with findings from captive studies which found that chimpanzees as young as 6 years of age demonstrated TPA. We argue that within-species variation in the expression of TPA, both in immatures and adulthood, provides evidence that the conflict management behaviours of young chimpanzees may be heavily influenced by social, ecological and demographic factors.

## 1. Background

Social conflict can be a negative consequence of group living, as increased gregariousness fosters greater potential for competition [1]. As a result, many species have developed strategies to mitigate conflict within their social group in order to offset its negative effects which can include the disruption of individual relationships [2] and/or group cohesion [3,4]. One form of conflict management involves the exhibition of affiliative behaviours in the aftermath of aggressive interactions. Post-conflict (PC) affiliation is well documented between former opponents in a variety of species (chimpanzees, *P. troglodytes* [5]; western gorillas, *Gorilla gorilla gorilla* [6]; bonobos, *P. paniscus* [7]; chacma baboons*, P. c. ursinus* [8]; hamadryas baboons, *Papio hamadryas ursinus* [9]; mandrills, *Mandrillus sphinx* [10]; bottlenose dolphins, *Tursiops truncates* [11]; spotted hyenas, *Crocuta crocuta* [12]; and goats, *Capra hircus* [13]), and may serve to reduce the arousal of the combatants, indicate that the conflict has ended, or re-establish affiliative relationships between former opponents [14].

One PC behaviour of particular interest is third-party affiliation (TPA), which is defined as affiliative contact given to recipients of aggression by uninvolved bystanders (regardless of initiation) [15]. This behaviour has been observed in a range of species [16–21]. While the function of this behaviour remains the subject of some debate (e.g. whether the intent of the third party is to console a victim of aggression or provide comfort to themselves via social interaction [22]), evidence from studies of great apes suggests that some functions of TPA may be prosocial in that they deliver a benefit to recent recipients of aggression. For example, work in captive bonobos (*P. paniscus*) demonstrates that unsolicited TPA reduces the victim's chances of receiving aggression from other group mates [23]. It is also possible that TPA, when given by a friend of the aggressor, serves to mend relationships between former opponents, as has been demonstrated in captive mandrills (*M. sphinx*) [21], wild baboons (*P. c. ursinus*) [9] and wild chimpanzees (*P. troglodytes*) [24–26]. Finally, unsolicited TPA has been shown to serve a conciliatory function in which third parties give support in what is often deemed an empathetic manner to victims of aggression, reducing the victim's anxiety and arousal following the aggressive event (captive chimpanzees, *P. troglodytes* [27–30]; wild chimpanzees, *P. troglodytes*: Tai, Côte d'Ivoire [24,26]; Mahale, Tanzania [5]; captive gorillas, *G. g. gorilla* [6,31]; and captive bonobos, *P. paniscus* [7,23,32]).

Notably, data on the ontogeny of third-party conflict management strategies in non-human primates are lacking, particularly in wild great apes. Existing evidence investigating third-party interventions suggests that as early as juvenility, some monkeys begin to act on behalf of others during agonistic encounters [33,34]. For example, juvenile Japanese macaques (*Macaca fuscata*) intervene in conflicts on behalf of their mothers, and give support based on knowledge of rank differences between their mother and her opponents [34]. Here, we investigate the ontogeny of TPA in wild chimpanzees in order to characterize TPA throughout the developmental period for the first time in a wild population.

The infant period in chimpanzees is characterized by high levels of maternal attachment [35] and lasts until approximately years 3–5 when weaning occurs [36] and offspring become nutritionally independent juveniles [37,38]. Chimpanzees experience an extended period of juvenility during which they continue to travel with their mothers until the onset of adolescence at 8–10 years old [37,38]. By adulthood, chimpanzees show responses to the emotions of others and engage in TPA with individuals who demonstrate signs of distress [5,24,28]. Notably, levels of TPA are highly variable between populations where it has been reported in the wild. For example, adult levels of TPA have been shown to vary considerably, occurring from as low as after 3.3% of conflicts [39] to as high as after 18.7% of conflicts [24]. A recent study of captive chimpanzees including individuals ranging in age from roughly 3.3 to 6.5 years found that juveniles expressed TPA, predominantly through play behaviours given to other immatures [40]. An earlier study also reported that juveniles as young as 6 years of age expressed TPA to all age classes and that levels were comparable to those of adults [27]. In general, it is difficult to make inferences about the developmental trajectories of wild chimpanzees from captive studies, as wild populations experience later maturation [41–46], perhaps resulting from a lack of provisioning. Furthermore, while captive studies provide important insight into the social and emotional capacities of chimpanzees as a species, it has been noted that captivity may impact chimpanzee social development [47,48], emotional expression [49], personality [47], and attention to social stimuli [50,51], making studies on wild populations a critical complement to that work. While captive studies have provided valuable insight into the presence of TPA in young chimpanzees, the extent and between-population variation of TPA in wild settings remains unknown.

In this study, we use 26 years of data to examine whether TPA (regardless of initiation and solicitation) occurs in wild immature chimpanzees of Gombe National Park, Tanzania. We also characterize TPA by mothers in the same dataset as an adult benchmark for interpreting immature TPA patterns. Immatures and mothers are likely to extend TPA at comparable frequencies due to their similar tendencies towards risk aversion. The cost of extending TPA to a recipient who may redirect aggression is likely to be heightened for both immatures who are of small body size and mothers with dependent offspring who are carrying or in close proximity to their vulnerable infants. Furthermore, mother--infant interactions can be used to assess the presence or absence of TPA in the population. For example, a study in Japanese macaques concluded that the study group did not demonstrate TPA after mothers failed to demonstrate affiliation towards offspring recipients of aggression [52].

In both immatures and mothers, we focus on PC grooming that has been reliably recorded for the duration of the long-term behavioural data collection, and has been shown to represent the most common form of TPA in adult chimpanzees [5,39,53]. In immatures we also examine play, a common measure of TPA in immature chimpanzees in captivity [27,40]. Since immaturity is considered an important period for the development of critical social skills [54], we hypothesize that wild chimpanzees will demonstrate TPA behaviour early in life. Because mothers represent the predominant social partner for young offspring [35], our analyses consider them separately from non-mothers to ensure that we do not mask patterns of TPA outside the mother--offspring relationship. First, we predict that immatures will groom and play with their mothers and non-mothers at frequencies greater than expected in the PC interval (less than or equal to 10 min following an aggressive event [39]) relative to randomly selected age-matched non-conflict intervals. We predict that as offspring age, the likelihood of grooming recipients of aggression in PC intervals will increase. However, as the frequency of play is known to decline with age, we predict a negative relationship between play as a TPA behavioural tool and age. Finally, we predict that mothers will give PC grooming to both offspring and non-offspring recipients of aggression.

## 2. Material and methods

### 2.1. Study population and data collection

We investigated the development of TPA (as measured by grooming and play) among immature wild chimpanzees at Gombe National Park, Tanzania. This population has been under continuous study since 1960, but our study focused on a 26-year interval (1988–2013) during which detailed behavioural and party composition data on immature (less than 12 years) individuals in the Kasekela community were available. Wild chimpanzees live in a fission–-fusion social system in which parties (subgroups) change in size and composition throughout the day. Within this dynamic system, mothers spend 40–70% of their time alone with their immature offspring [55,56]. Data on immature individuals and their mothers were collected via Family Follows during which well-trained Tanzanian field assistants and researchers target a focal family (mother, infant and next youngest offspring) and record the behaviour for each focal family member at 1 min instantaneous point samples [57]. Partner identity is recorded for any social interaction, including grooming and play. Additionally, party composition is recorded throughout the follow, at 5 min intervals until 2011 and 15 min intervals from 2011 to 2013. The target duration of Family Follows varied from 6 to 12 h (night nest-to-night nest) over the course of the study, with the goal to follow each mother–infant pair in the community once per month.

Family Follow data also include detailed information on aggressive events, which are recorded opportunistically on all members of the party. We extracted these aggressive events, defined as any threat, directed display, chase or attack between two or more chimpanzees in the group [35]. Most aggressive encounters are loud and conspicuous events, facilitating a reliable record of conflicts involving both focal and non-focal individuals [58]. For each event, we identified the aggressor and recipient, though we did not code interactions as being ‘won’ or ‘lost’. For the purpose of our analyses, events involving multiple aggressors were included as one event from the recipient's perspective, and events involving multiple recipients were separated into dyads. For example, if individual FR chased and hit individuals GA and GM, two aggressor–recipient dyads (FR-GA and FR-GM) were coded. We determined multiple aggressive interactions between a particular dyad to be a single event if two successive interactions occurred within 10 min of each other (following [59]), and only included data from the last event in the analysis. The end of the aggressive interaction marked the start of the 10 min PC interval. Aggressive events included in the immature TPA analyses included only those in which an immature was a bystander present in the party, and not involved in the aggression. Aggressive events in the mother TPA analyses included those in which a mother was a bystander in the party, and not involved in the aggression. Aggressive events included aggressors and recipients of all age and sex classes.

### 2.2. Analyses

To investigate how TPA varies across development, we compared grooming and play frequencies from bystanders to recipients of aggression in the PC interval to grooming and play frequencies between individuals in random 10 min non-conflict intervals. Non-conflict intervals were those in which an immature or a mother was observed for a consecutive 10 min period that was not preceded by aggression. To control for factors that could influence grooming or play given by immatures and grooming given by mothers in both PC and non-conflict intervals, we labelled all PC and non-conflict intervals based on categorical party size and feeding context. Intervals that began with party size less than 5 individuals were labelled small, while those with a party size greater than or equal to 5 individuals were labelled large (following [59]). Furthermore, due to expected low levels of grooming and play during feeding periods, we labelled all intervals as ‘feeding’ if an immature or mother was recorded as feeding on the first minute or minute prior to the start of the interval.

To ensure that PC interactions were captured reliably, we restricted analyses to focal data. Though direction of grooming and play was recorded in our long-term dataset, which individual initiated the behaviour was not regularly recorded; thus we were unable to differentiate between solicited and unsolicited interactions. No immatures were observed to groom either mothers or non-mothers in PC periods until after 1.5 years of age. Thus, we divided the immature period into three age bins that correspond roughly to chimpanzee life-history stages: infancy (1.5–5 years), juvenility (5–8 years) and adolescence (8–12 years). Captive studies often employ matched-control methods in which non-conflict data are collected when the third party and the recipient are together and under similar conditions as the original aggressive event [27,40]. Owing to the fission–fusion social system of chimpanzees and our limited sample size of TPA events, our ability to compile reliable matched-control samples between specific third parties and recipients of aggression was limited. Thus, these analyses are not individual based, but rather pool data from all immatures of the same age category together across the duration of the study. This approach does not assess individual tendencies towards TPA, but determines whether the probability of TPA towards a class of individuals (mothers/non-mothers) is higher in PC periods than would be expected of general patterns of grooming and play in non-conflict periods for immatures of a given age category.

To account for availability of grooming and play partners, we analysed only non-conflict intervals where the immature was with his/her mother, or non-conflict intervals where the immature was with at least one non-mother. To understand levels of TPA given by mothers, we followed a similar approach of comparing grooming frequencies in the PC interval to grooming frequencies in non-conflict intervals, considering grooming given to offspring of any age versus non-offspring separately in our analyses. Again, to account for availability of grooming partners, we analysed only non-conflict intervals where the mother was with an immature offspring, or non-conflict intervals where the mother was with at least one non-offspring.

We used a Monte Carlo randomization approach with stratified sampling to test whether immatures groomed or played with the recipients of aggression more frequently in the PC interval than in random 10 min intervals that did not follow a conflict. Specifically, we resampled with replacement random 10 min non-conflict intervals from follows of immatures in a given age bin. To ensure we did not include portions of PC intervals while sampling non-conflict intervals, all PC intervals were removed from the dataset before sampling of non-conflict intervals took place. The number of 10 min non-conflict intervals randomly selected for re-sampling always matched the number of PC intervals for a given recipient type (e.g. mother/non-mother) in a given age bin. We stratified the non-conflict sample based on the number of PC intervals in a given feeding context (Y/N) and at a given categorical party size (small/large) (tables 1 and 2). For example, if there were 100 PC intervals where the mother was the recipient of aggression from ages 1.5 to 5 years and 25 occurred in a feeding context and small parties, then 25 of the 100 randomly selected non-conflict intervals were drawn from feeding contexts in small parties. From each stratified random sample, we calculated the proportion of non-conflict intervals where grooming or play occurred. This process was repeated for 10 000 iterations to create distributions of the proportion of non-conflict intervals with grooming or play towards a given recipient type in each immature age class. We then compared the proportion of PC intervals containing immature grooming towards or playing with a mother or a non-mother who was the recipient of aggression to the distribution of non-conflict intervals containing grooming towards or playing with a mother or a non-mother. PC grooming and PC playing were considered significantly different from non-conflict grooming or non-conflict playing if the PC proportion fell within or beyond the outer 2.5% ends of the distribution of non-conflict proportions. The same method was used to compare PC grooming given by mothers towards offspring and non-offspring to general grooming patterns. All randomization testing was performed in R (v. 3.0.1, R Core Development Team 2013) using the ade4 package and as.randtest function to determine *p*-values.

**Table 1.** Number of aggression events and the proportion of events with grooming and play in each age category for analyses investigating immature grooming of mothers after they were recipients of aggression. PC, post-conflict. Numbers in parentheses = proportions of total PC intervals in which grooming or play was directed by the immature to their mother.

age category	*N* aggression events witnessed	*N* PC intervals with grooming	bootstrapped mean proportion of non-conflict periods with grooming	*N* PC intervals with play	bootstrapped mean proportion of non-conflict periods with play
1.5–5.0	457	17 (0.037)	0.026	15 (0.033)	0.026
5.0–8.0	180	7 (0.039)	0.059	1 (0.006)	0.002
8.0–12.0	185	7 (0.038)	0.051	0 (0)	0.0008

**Table 2.** Number of aggression events and the proportion of events with grooming and play in each age category for analyses investigating immature grooming of non-mothers after they were recipients of aggression. PC, post-conflict. Numbers in parentheses = proportions of total PC intervals in which grooming or play was directed by the immature to a non-mother.

age category	*N* aggression events witnessed	*N* PC intervals with grooming	bootstrapped mean proportion of non-conflict periods with grooming	*N* PC intervals with play	bootstrapped mean proportion of non-conflict periods with play
1.5–5.0	647	3 (0.005)	0.031	41 (0.063)	0.173
5.0–8.0	378	4 (0.011)	0.076	16 (0.042)	0.102
8.0–12.0	284	0 (0)	0.068	3 (0.011)	0.049

## 3. Results

Our data included 2131 aggressive interactions that occurred when immatures aged 1.5–12.0 years were present in the party (see tables 1 and 2 for summaries by immature age bin). The majority of aggression observed in this study involved an adult recipient (62%), while 38% of events involved an immature recipient. There were 38 instances when an immature groomed the recipient of aggression, 31 of which were directed towards mothers. The remaining PC grooming events were directed towards maternal siblings (*N* = 2) with 5 directed towards individuals that were unrelated through the matriline (*N* = 2 events towards non-maternally related immatures; *N* = 3 events towards non-maternally related adults).

There were 76 instances when an immature played with the recipient of aggression, 16 of which were directed towards mothers. The remaining PC play events were directed towards maternal siblings (*N* = 13) with 47 directed towards individuals that were unrelated through the matriline (*N* = 36 events towards non-maternally related immatures; *N* = 11 events towards non-maternally related adults).

There were 2571 aggressive interactions that occurred when mothers with dependent offspring were present in the subgroup. The mother groomed the recipient of aggression in 67 instances, 49 of which were directed towards offspring (ranging in age from 1.7 to 16.9 years). The remaining PC grooming events were directed towards adult maternal siblings (*N* = 6), non-maternally related immatures (*N* = 1) and non-maternally related adults (*N* = 11). In 1569 aggressive interactions, the recipient was an adult and not an offspring of the focal mother; thus, mothers given grooming to peers who received aggression following only 0.01% of all conflicts.

### 3.1. Third-party affiliation directed towards mothers and non-mothers by immatures

In all age bins, immatures did not groom mothers who received aggression in PC intervals greater than expected based on random non-conflict intervals (ages 1.0–5.0, *p* = 0.134; ages 5.0–8.0, *p* = 0.271; ages 8.0–12.0, *p* = 0.506). Immatures did not play with mothers who received aggression in PC intervals greater than expected based on random non-conflict intervals at ages 1.5–5.0 (*p* = 0.469) and ages 8.0–12.0 (*p* = 1). Immatures played with mothers who received aggression in PC intervals greater than expected based on random non-conflict intervals at ages 5.0–8.0 (*p* = 0.034); however, this significant result is based on a single instance of an immature playing with their mother during a PC period (table 1; electronic supplementary material, figure S1*a,b*).

Grooming of non-mother recipients of aggression in PC intervals happened significantly less than expected based on non-conflict grooming patterns for all age bins (ages 1.5–5.0, *p* < 0.0005; ages 5.0–8.0, *p* < 0.0002; ages 8.5–12.0, *p* < 0.0002). Playing with non-mother recipients of aggression in PC intervals happened significantly less often than expected based on non-conflict playing patterns for all age bins (ages 1.0–5.0, *p* = 0.0009; ages 5.0–8.0, *p* = 0.0003; ages 8.0–12.0, *p* = 0.0005; table 2; electronic supplementary material, figure S2*a,b*).

### 3.2. Third-party affiliation directed towards offspring and non-offspring by mothers

Mothers exhibited TPA towards their offspring. We found that mothers groomed offspring recipients of aggression in PC intervals at frequencies that were significantly higher than expected of random non-conflict intervals (*p* < 0.0009). However, mothers groomed non-offspring recipients of aggression in PC intervals at frequencies that were significantly lower than expected of random non-conflict intervals (*p* < 0.0009) (table 3; electronic supplementary material, figure S3).

**Table 3.** Number of aggression events and the proportion of events with grooming for analyses investigating mothers grooming offspring and non-offspring after they were recipients of aggression. PC, post-conflict. Numbers in parentheses = proportions of total PC intervals in which grooming was directed by the mother to an offspring or non-offspring.

relationship to recipient	*N* aggression events witnessed	*N* PC intervals with grooming	bootstrapped mean proportion of non-conflict periods with grooming
offspring	553	49 (0.089)	0.034
non-offspring	2018	18 (0.009)	0.077

## 4. Discussion

In this study, we provide the first investigation of the development of TPA in wild chimpanzees and compared it to TPA patterns in mothers with dependent offspring in the same community as a benchmark for TPA patterns in adults. Overall, our results demonstrate that while mothers do express TPA towards their offspring, immature chimpanzees at Gombe National Park do not express TPA to mothers as measured by grooming between the ages of 1.5 and 12.0 years, and very limited evidence that they expressed TPA via play (table 4). Furthermore, neither mothers nor immatures expressed TPA towards non-offspring or non-mothers, respectively. Specifically, we predicted that immatures of all age categories would groom and play with mothers who were the recipients of aggression more in PC intervals than non-conflict intervals. Instead, we found that immatures in our population did not give grooming to mothers who received aggression more frequently than expected based on non-conflict grooming patterns in any age category. We also found limited evidence that immatures played with mothers who received aggression more frequently than expected based on non-conflict play patterns; only age category 5.0–8.0 yielded a significant result; however, this was based on a single instance of playing between an immature and their mother and thus should be interpreted with caution as it likely represents a false positive result. Furthermore, immatures groomed and played with non-mothers significantly less in PC intervals compared with non-conflict intervals. Interestingly, mothers did not groom non-offspring in PC intervals greater than expected based on general patterns. However, mothers did extend TPA to offspring during PC periods more frequently than expected based on non-conflict grooming patterns. It remains possible that TPA behaviour, at least to the extent that mothers to exhibit TPA towards closely related individuals, develops in full by adulthood or is expressed in other ways (e.g. approaches, touches, embraces, etc.).

**Table 4.** Summary of results on immature grooming and play of mother and non-mother recipients of aggression in each age category. X denotes no significant difference between PC proportion and bootstrapped mean proportion of non-conflict intervals with grooming or play. Symbol ‘>’ denotes PC proportions significantly greater than bootstrapped mean proportion of non-conflict intervals with grooming or play. Symbol ‘<’ denotes PC proportions significantly lower than bootstrapped mean proportion of non-conflict intervals with grooming or play.

	with mothers		with non-mothers	
age category	grooming	play	grooming	play
1.5–5.0	X	X	<	<
5.0–8.0	X	>	<	<
8.0–12.0	X	X	<	<

Intriguingly, our study suggests limited evidence for TPA in either immatures or mothers. We suggest that TPA may be constrained in immatures and mothers with dependent offspring due to the potential risks that surround aggression. Agonistic behaviours are risky for the perpetrator, the recipient and third parties in that involvement may result in social exclusion, anxiety, injury or death [2,60,61]. Lower-than-expected levels of grooming and play towards non-mothers in PC intervals suggest that immatures avoid grooming non-maternal recipients of aggression, indicating that the benefits of giving support to unrelated individuals do not outweigh the risks of doing so in a wild population. Small body size [62] and low rank may give immatures limited social power [3] to give affiliation following conflicts, and heighten the risk of physical harm. Mothers with dependent offspring may face similarly elevated risks due to the presence of dependent offspring. It is possible that giving affiliation to unrelated recipients of aggression is less likely in a wild population where fluid subgrouping patterns may allow mothers and immatures to simply leave high-risk groups after an aggressive event or avoid them altogether.

The rarity of TPA in Gombe immatures complements a similar developmental study from captive chimpanzees that found limited evidence for TPA prior to age 6 years [40]. Immatures in that captive population [40] expressed TPA mainly in the form of social play, arm's length proximity, and sitting in contact, and was given only to other immatures although adult–adult conflicts were excluded from analyses. The lack of evidence for TPA in later age classes at Gombe contrasts with one captive study that found TPA to be fully expressed at adult levels in individuals as young as 6 years of age [27]. The differences between the developmental trajectories of captive versus wild populations [41–46] call for additional research from wild populations in order to elucidate the impact of demography and grouping patterns on TPA in natural settings. Furthermore, as it is possible that TPA is expressed differently in captivity versus the wild, it is necessary to explore alternative forms of PC affiliation in immature chimpanzees including proximity, approaches, touches and embraces, which have been included in previous captive studies [27,40]. Cross-site comparisons are necessary to determine whether the lack of TPA expressed by Gombe immatures is representative of population differences, or a broader trend in wild chimpanzees.

At Gombe, mothers only gave TPA to their own offspring. This result could be explained by the behaviour of the offspring following aggression, as it is not uncommon for young individuals to run to their mothers following potentially dangerous or stressful events. Alternatively, this result may highlight the possibility that TPA at Gombe remains a rare behaviour even in adulthood. Variation of TPA during development and in adulthood [24,39] suggests that this behaviour may be expressed according to differences in social environment and/or ecology. It is possible that TPA plays a limited role in the conflict management repertoire of this population. More broadly, grooming behaviour in chimpanzees has been shown to be influenced by culture [63], raising the intriguing possibility that group-specific tendencies shape the expression of TPA. Future work will examine the expression of TPA in other adults of the Gombe population including adult males and non-mothers to determine group levels of TPA, as well as test hypotheses for the function of this behaviour.

While our study does not explicitly test the function of TPA, evidence from prior studies in chimpanzees indicates the behaviour may be prosocial as it reduces future aggression, anxiety and arousal in recipients of aggression [27–33]. Interestingly, in infancy human children are attentive to the negative emotions of others [64–66], and begin to show both helping and comforting behaviours towards parties in distress in their second year of life [67,68]. The early emergence of helping, cooperation and conflict management in human development suggests a biological basis to these behaviours that may be altered later based on culture [69], which may vary across populations. To fully understand when this biological predisposition emerged in our evolution, it is critical to understand patterns of similar behaviours across development in our great ape relatives*.* Here, we provide the first investigation of the development of TPA in wild chimpanzees, a behaviour that may be prosocial due to the benefits it carries for recent recipients of aggression. However, chimpanzees represent just one of the two extant species of *Pan*. A recent study of sanctuary bonobos revealed that juveniles as young as age 3 years were more likely to give TPA to recipients of aggression compared to adults, and that mother-reared individuals were more likely to give TPA than orphans [32,70]. Notably, adult bonobos are characteristically described as being more egalitarian in nature than chimpanzees [71]. Future studies are needed to clarify the point at which TPA emerges in wild bonobos and whether the two species of *Pan* differ in their prosocial predispositions.

## 5. Conclusion

Conflict management strategies are a salient feature of group-living species. While studies often focus on adult patterns, it is also important to consider if and how conflict management behaviours develop; however, few studies have documented their ontogeny. Here, we found that, while mothers exhibit offspring-biased TPA, immature chimpanzees in our study community do not give TPA to either mothers or non-mothers. The lack of TPA during development and documented variation between study sites in levels of both immature and adult TPA suggest that the cooperative, conflict-mitigating behaviours of young chimpanzees are heavily influenced by social, ecological and demographic factors. The within-species plasticity of these behaviours is intriguing and future comparative studies are necessary to determine how ecological and social factors relate to the existence and prevalence of conflict management strategies such as TPA.

## Supplementary Material

Subjects Biographical Information & Sample Sizes

## Supplementary Material

Immature grooming and playing with mothers in post-conflict versus non-conflict intervals

## Supplementary Material

Immature grooming and playing with non-mothers in post-conflict versus non-conflict intervals

## Supplementary Material

Mother grooming of offspring and non-offspring in post-conflict versus non-conflict intervals

## Data accessibility

All data on PC aggression are reported in this paper. Data used to bootstrap proportion of grooming in non-conflict intervals are available on Dryad: http://dx.doi.org/10.5061/dryad.70c9v [72].
