# Supplementary material for: Limited evidence for third-party affiliation during development in wild chimpanzees (Pan troglodytes schweinfurthii)
Source: R Soc Open Sci. 2017 Sep 13;4(9):170500. doi: 10.1098/rsos.170500 (PMC5627097; doi:10.1098/rsos.170500)
Supplement: Subjects Biographical Information & Sample Sizes [file rsos170500supp1.docx]

**Limited Evidence for Third Party Affiliation During Development in Wild Chimpanzees (*Pan troglodytes schweinfurthii*)**

Jordan A. Miller^a*^, Margaret A. Stanton^a^, Elizabeth V. Lonsdorf^b^, Kaitlin R. Wellens^a^, A. Catherine Markham^c^, & Carson M. Murray^a^

| **Focal Immature ID** | **Sex** | **Age Categories Included** | **# PC Interactions** |
| --- | --- | --- | --- |
|  |  |  |  |
| BRZ | M | 1.5 - 5.0 | 1 |
| DIA | F | 5.0 - 8.0 | 1 |
| ERI | M | 1.5 - 5.0 | 12 |
| FAD | F | 1.5 - 5.0 | 1 |
| FAM | F | 5.0 - 8.0 | 2 |
| FE | M | 1.5 - 5.0, 5.0 - 8.0 | 4 |
| FFT | M | 1.5 - 5.0 | 2 |
| FLI | F | 1.5 - 5.0 | 10 |
| FND | M | 1.5 - 5.0, 5.0 - 8.0 | 11 |
| FO | M | 1.5 - 5.0 | 3 |
| FS | F | 1.5 - 5.0, 5.0 - 8.0 | 2 |
| FU | M | 1.5 - 5.0, 5.0 - 8.0 | 6 |
| GA | F | 1.5 - 5.0, 5.0 - 8.0 | 10 |
| GD | M | 1.5 - 5.0, 8.0 - 12.0 | 11 |
| GIM | M | 5.0 - 8.0 | 1 |
| SAF | F | 1.5 - 5.0 | 1 |
| SDB | M | 1.5 - 5.0 | 1 |
| SL | M | 8.0 - 12.0 | 1 |
| SN | M | 1.5 - 5.0 | 5 |
| SR | F | 1.5 - 5.0, 5.0 - 8.0 | 5 |
| TAB | F | 1.5 - 5.0, 5.0 - 8.0 | 5 |
| TG | F | 1.5 - 5.0 | 2 |
| TN | M | 1.5 - 5.0, 5.0 - 8.0 | 6 |
| TOM | M | 8.0 - 12.0 | 2 |
| TZN | M | 1.5 - 5.0 | 1 |
| ZEL | F | 8.0 - 12.0 | 3 |
| ZIN | M | 1.5 - 5.0 | 5 |
|  |  |  |  |

**SI, Table 1.** Focal immature subjects and TPA sample sizes included in the analyses.
