# Supplementary material for: Limited evidence for third-party affiliation during development in wild chimpanzees (Pan troglodytes schweinfurthii)
Source: R Soc Open Sci. 2017 Sep 13;4(9):170500. doi: 10.1098/rsos.170500 (PMC5627097; doi:10.1098/rsos.170500)
Supplement: Immature grooming and playing with non-mothers in post-conflict versus non-conflict intervals [file rsos170500supp3.docx]

**Limited Evidence for Third Party Affiliation During Development in Wild Chimpanzees (*Pan troglodytes schweinfurthii*)**

Jordan A. Miller^a*^, Margaret A. Stanton^a^, Elizabeth V. Lonsdorf^b^, Kaitlin R. Wellens^a^, A. Catherine Markham^c^, & Carson M. Murray^a^

**
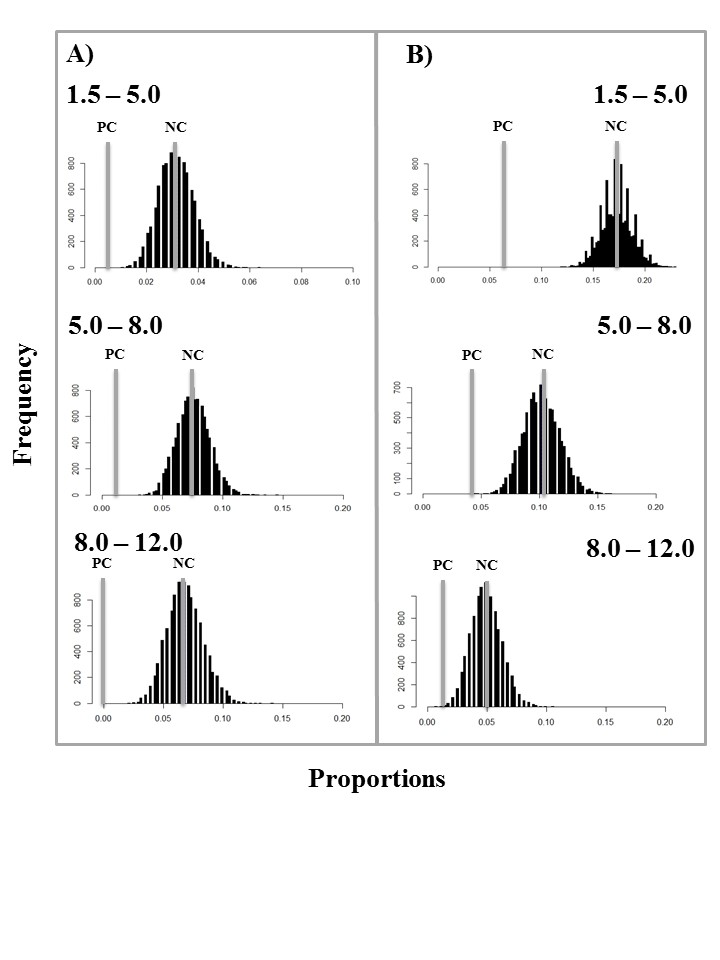
**

**SI, Figure 2.** Histogram of 10,000 proportions of non-conflict intervals in which an immature A) groomed or B) played with a non-mother obtained from a randomization procedure for individuals of age class 1.5 – 5.0, 5.0 – 8.0, 8.0 – 12.0. PC = Average proportion of PC intervals in which the non-mother was groomed or played with. NC = Bootstrapped mean of proportions in which the non-mother was groomed or played with in a random non-conflict interval.
