# Supplementary material for: Limited evidence for third-party affiliation during development in wild chimpanzees (Pan troglodytes schweinfurthii)
Source: R Soc Open Sci. 2017 Sep 13;4(9):170500. doi: 10.1098/rsos.170500 (PMC5627097; doi:10.1098/rsos.170500)
Supplement: Mother grooming of offspring and non-offspring in post-conflict versus non-conflict intervals [file rsos170500supp4.docx]

**Limited Evidence for Third Party Affiliation During Development in Wild Chimpanzees (*Pan troglodytes schweinfurthii*)**

Jordan A. Miller^a*^, Margaret A. Stanton^a^, Elizabeth V. Lonsdorf^b^, Kaitlin R. Wellens^a^, A. Catherine Markham^c^, & Carson M. Murray^a^

**
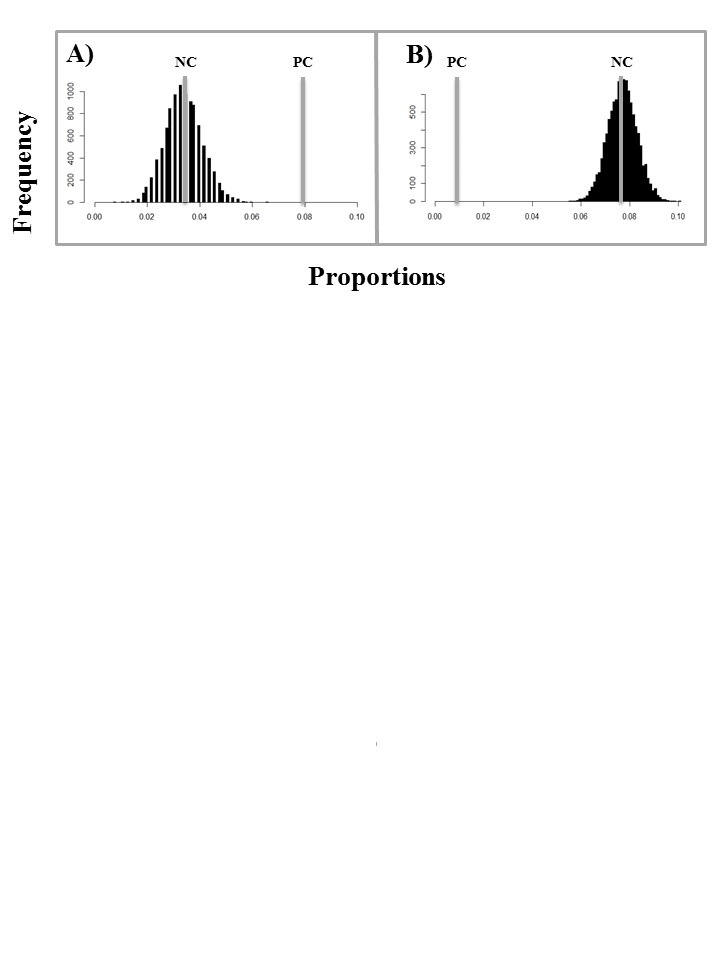
**

**SI, Figure 3.** Histogram of 10,000 proportions of non-conflict intervals in which mothers groomed A) offspring and B) non-offspring. PC = Average proportion of PC intervals in which an offspring or non-offspring was groomed. NC = Bootstrapped mean of proportions in which an offspring or non-offspring was groomed in a random non-conflict interval.
